# Clinical utility of a non-invasive urine test for risk assessing patients with no obvious benign cause of hematuria: a physician-patient real world data analysis

**DOI:** 10.1186/s12894-018-0327-6

**Published:** 2018-03-09

**Authors:** Tony Lough, Qingyang Luo, Carthika Luxmanan, Alastair Anderson, Jimmy Suttie, Paul O’Sullivan, David Darling

**Affiliations:** 1 Pacific Edge Limited, 87 St David Street, Dunedin, 9016 New Zealand; 2 University of Otago, Dunedin, New Zealand; 3 Merck, Sharpe & Dohme, Auckland, New Zealand

**Keywords:** Asymptomatic microscopic hematuria, Biomarker, Clinical parameters, Clinical utility, Cystoscopy, Diagnostic, Hematuria, Molecular diagnostic, Risk assessment, Urothelial carcinoma

## Abstract

**Background:**

The non-invasive Cxbladder urine test system has demonstrated clinical utility in ruling out urothelial carcinoma (UC) in patients with asymptomatic microscopic hematuria (AMH), suggesting that the number of invasive diagnostic tests, including cystoscopy, used in this patient population may be reduced by Cxbladder testing prior to conducting a full urological work-up. The aim of this study was to demonstrate the enhanced clinical utility of communicating objective information on diagnostic decisions made by individual physicians on individual patients with AMH.

**Methods:**

Three hundred ninety-six physician-patient decisions were generated from twelve participant physicians evaluating real world case notes from the same 33 patients presenting with AMH. Each physician reviewed and recommended diagnostic tests and procedures based on each patient’s referral data and then re-evaluated their clinical recommendation following disclosure of the non-invasive Cxbladder urine test result. Changes assessed were the total number of requested diagnostic procedures and the number of invasive procedures, including cystoscopy, following addition of information from Cxbladder in the Triage and Triage and Detect modalities.

**Results:**

Physicians made significant changes to their diagnostic behavior for patients with AMH when presented with Cxbladder test results, including a reduction in the number of total and invasive procedures including cystoscopy for individuals identified as having a low probability of UC. The intensity of investigation was targeted and increased, including use of total procedures and cystoscopy, for patients identified by Cxbladder tests as having a high probability of UC: urologists increased the level of investigation for both total procedures and invasive procedures. The outcome resulted in patients with a high risk of UC receiving appropriate guideline-recommended invasive diagnostic tests. Patients who tested negative were offered fewer and significantly less invasive procedures. This change in physician behavior results in an increased clinical and patient utility, lower risk of missed UC and invasive test-related harm incidents.

**Conclusions:**

This study demonstrated the potential for increased clinical resolution and significantly enhanced patient management, when physicians consider Cxbladder test results in their clinical evaluation. The change in physician behavior led to more appropriate diagnostic procedure selection and resource allocation to the benefit of both patients and healthcare systems.

**Electronic supplementary material:**

The online version of this article (10.1186/s12894-018-0327-6) contains supplementary material, which is available to authorized users.

## Background

Hematuria, or blood in the urine, is a common occurrence in clinical practice. Asymptomatic microscopic hematuria (AMH; defined as > 3 red blood cells/high-powered field in a properly collected urine sample) is present in up to 24% of the general population [1] and is the primary reason for more than 485,000 referrals to urologists in the US every year [2].

The current American Urological Association (AUA) guidelines recommend a full urological work-up to diagnose or rule out UC in patients with AMH within 180 days [3, 4], however several barriers to referral for a full urological work-up exist. For example, clinical diagnostic algorithms for patients with hematuria are complicated, difficult to follow and enable a degree of latitude in their interpretation [1]. Many physicians are also conscious of the burden of invasive procedures and the potential for harm and there is a lack of compliance by patients when confronted with the prospect of many and varied invasive procedures. Invasive procedures, such as cystoscopy and contrast computed tomography (CT) scans, have the potential to impact their patients in terms of adverse events, financial cost and emotional impact [1, 4–12]. As such, guideline recommendations to administer full urological work-ups to all patients presenting with hematuria may only be selectively adhered to [12, 13].

In particular, as few as 10% of women with hematuria are referred to urologists [14]. Likewise, < 14% of patients with hematuria undergo cystoscopy or radiological investigation within 180 days and a recent Australian study reported delays of up to 1165 days between initial presentation of hematuria and subsequent treatment for UC [15, 16]. This is particularly concerning given that delayed diagnoses are known to negatively influence outcomes for patients with UC with every day increasing the risk of death for a patient with UC by 1% [17].

The high probability of delayed treatment is particularly evident for younger and female patients. Treatment for UC is more likely to be delayed for women than men, and although the incidence of UC is higher for men [18–23], women are less likely to undergo procedures after being referred and have a higher risk of presenting with UC at an advanced stage and have poorer survival outcomes [21, 24]. Therefore, a simple, rigorous and accurate segregation of patients using a non-invasive risk-assessment tool may reduce barriers to referral and focus the intensity and prioritisation of patients for workup.

The need for such a non-invasive UC risk-assessment tool for patients with hematuria contrasts with skepticism regarding the clinical utility of urine biomarkers and molecular diagnostics in diagnosing UC, particularly predictions that routine use of urine biomarker tests will ultimately increase the use of invasive diagnostic procedures [1, 15, 25].

The non-invasive Cxbladder urine test can be used to identify patients with hematuria with a low or high probability of UC in its Triage (sensitivity 0.95; negative predictive value 0.98) and Detect (specificity 0.85; sensitivity 0.82) modalities, respectively [26, 27]. Cxbladder has previously demonstrated population-level clinical utility in reducing the net number of diagnostic tests using real world clinical data in a retrospective evaluation of participating urologist’s decisions. [12]. Specifically, invasive procedures, requested by urologists assessing a real-world sample population of patients with AMH [12] led to all patients ultimately diagnosed with UC receiving a cystoscopy and/or CT scan as part of a diagnostic work-up, in line with the AUA guidelines, [4, 12]. There is therefore potential for an increased risk of harm for the one-third of UC-positive cases potentially missed using non-invasive procedures in the baseline case.

Having demonstrated that Cxbladder may decrease the use of invasive tests across a population of patients with AMH, the aim of the present study was to provide greater resolution by investigating the potential change in clinical utility from communicating objective information from Cxbladder tests on the diagnostic decisions made by individual physicians for individual patients with AMH.

## Method

### Participant and patient case details

Full methodological, case and participating physician details were reported in Darling et al. (2017) [12]. Briefly, patients were prospectively recruited and systematically selected to represent a broad patient demographic and clinical spectrum. Three hundred ninety-six physician-patient clinical decisions were evaluated where 12 urologists (participant physicians; Additional file 1: Table S1) with experience in the use and application of Cxbladder in their clinical settings were recruited. Participating physicians were offered honoraria as compensation for the time spent participating in the study. Experience in the use and application of Cxbladder was defined as using Cxbladder more than 10 times in real-world situations. Each participant physician individually evaluated the same 33 case-note clinical referral data from patients presenting with AMH systematically selected from the database of patients enrolled in previous prospective clinical studies of Cxbladder. Patients consented to the anonymous use of their urine sample and clinical information, IRB approval details are presented below.

All patients met the AUA definition of AMH with the possibility of UC requiring a urological work-up, including invasive diagnostic procedures [3]. Each participant physician was asked to review and recommend diagnostic procedures for each case based on the patient’s evaluation from normal referral data. Participant physicians were asked to select a set of urological investigation procedures from a list of investigation procedures recommended in the AUA guidelines [3]. For the purposes of this study and to enable the reduction of any systemic bias, participant physicians were instructed to consider all tests and procedures to be fully funded, with no additional cost incurred by the patient. Participant physicians were not informed of the final UC status of each case.

Participant physicians performed further recommended clinical evaluations for each case twice, firstly in the context of the information derived from Cxbladder Triage alone and secondly when both Cxbladder Triage and Detect were presented. The data format was consistent with the commercially available Cxbladder tests, including a report outcome, explanation of the Cxbladder test methodology and guide to interpreting the results [26, 27]. Outcomes were defined as low probability of UC, ‘negative’, or standard clinical workup, ‘physician-directed protocol’, for Cxbladder Triage and ‘normal’, ‘elevated’ or ‘high’ gene expression for Cxbladder Detect. Elevated and high results in the Detect modality were presented as ‘positive’ for UC for the purposes of this study.

### Study endpoints

The co-primary endpoints were changes in the total number of diagnostic procedures used over all patient cases and the number of invasive procedures requested following disclosure of diagnostic information from Cxbladder Triage and Cxbladder Triage and Detect. For the purposes of this study, cystoscopy (flexible or rigid) and CT scans (contrast or non-contrast) were defined as invasive diagnostic procedures whereas ultrasound, urine cytology and UroVysion® fluorescence in situ hybridization were defined as non-invasive procedures and tests.

### Statistical procedures

A random-effect linear mixed model was used for net change analyses for total procedures, invasive procedures and each individual procedure requested, for each physician-patient interaction. Fixed effects were time (baseline, after test) and test results, and random effects were patient and participant physician. The average number of procedures per interaction at baseline was calculated for total procedures, invasive procedures and each individual procedure. 95% confidence intervals (CI) that did not include zero were considered to be statistically significant (*p* < 0.05).

Transition probability analyses were performed for individual invasive procedures. Transition probabilities with a denominator ≥10 and an upper limit of its CI ≥ 0.5 were considered to be statistically significant. If the upper and lower limits of the CI were ≥0.5, then the transition probability was considered to be highly statistically significant (*p* < 0.01).

### Heatmap data graphic

Data were graphically presented as heatmap calculations to aid the visualisation of the results at the individual physician-patient interaction level. The heatmaps show the range of variation in decision making and the change in decisions with and without the addition of the Cxbladder result. The baseline heatmaps provide the total count of procedures at each of the 396 physician-patient interactions in the absence of availability of the Cxbladder result. The change, relative to this baseline, made when the Cxbladder result was disclosed reflects the change in physicians decision making. Heatmap columns represent individual physicians and rows represent individual patients with each row and column intersection becoming a physician-patient interaction. Patients (rows) are grouped by Cxbladder result with colour bar linkage, patient ID and test result. The baseline heatmaps include the actual number of procedures per interaction and the associated change heatmap includes the change relative to baseline per interaction. The colour code in the heatmap is used to indicate extent of the change in the number of tests and procedures used in the physician-patient interaction. An increase in the change is represented in shades of red or a decrease in the change is represented in shades of green or no change relative to baseline is in white. The greater the intensity of colour the greater the change in decisions.

## Results

**Fig. 1:**
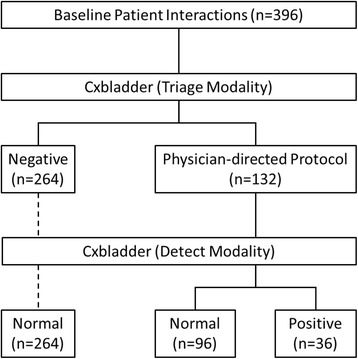
Statement for Reporting Diagnostic Accuracy (STARD) Diagram illustrating the diagnostic cascade for patient interactions. All patients that tested Cxbladder Triage 'negative' also tested Cxbladder Detect 'normal'

Each participant physician evaluating an individual patient represents an independent physician-patient interaction. Overall, 792 post-baseline decision nodes were generated after 396 baseline physician-patient clinical decisions were made following the provision of firstly Cxbladder Triage followed by Cxbladder Detect data (Fig. 1). At the first decision node, 264/396 clinical decisions (66.7%) tested negative for Cxbladder Triage. No cases where a test was reported as negative for Cxbladder Triage subsequently tested positive using Cxbladder Detect. Of the 132 clinical decisions where a physician-directed protocol was recommended by Cxbladder Triage, 36 (27.3%) carried positive results following Cxbladder Detect.

**Fig. 2:**
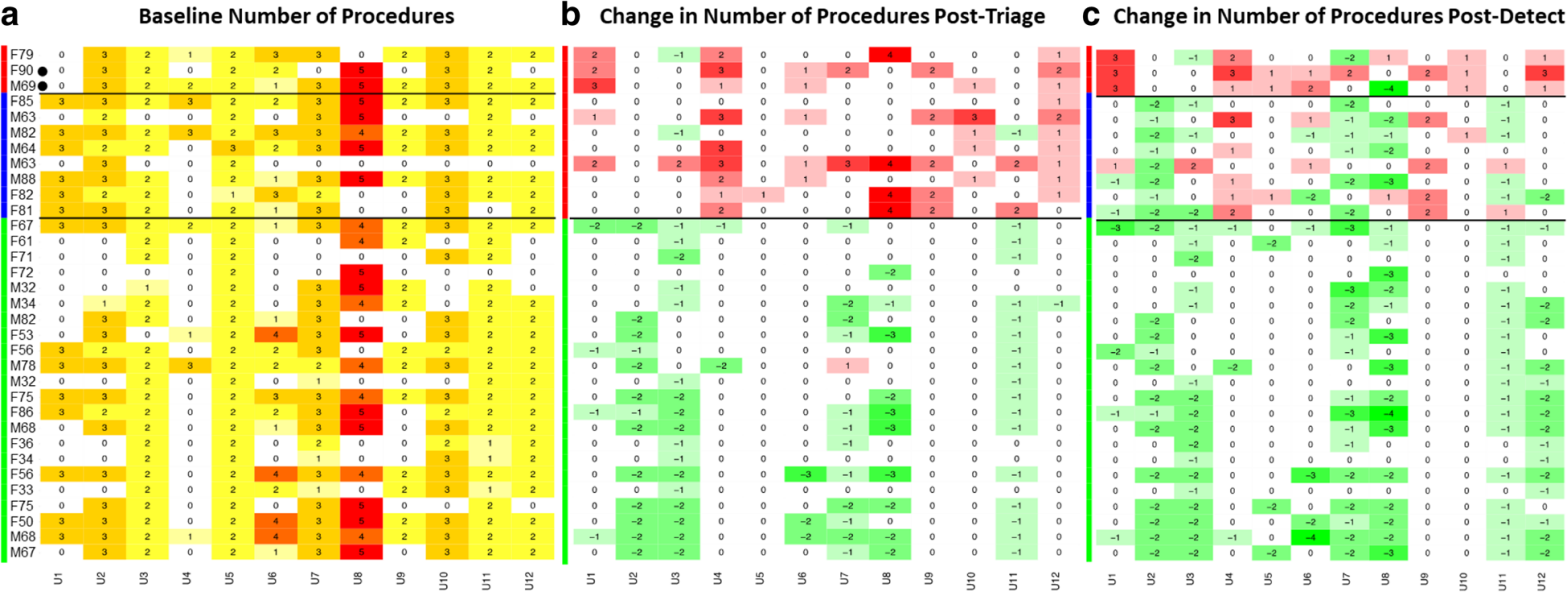
Heat maps representing the total number of diagnostic tests. Panel **a**: Baseline number of procedures; Panel **b**: Change from baseline after presenting the results of Cxbladder Triage; Panel **c**: Change from baseline after presenting the results of Cxbladder Triage and Detect. Columns represent participant physicians. Rows represent patients. Each cell represents a patient-physician decision node. Reds represent decisions nodes with added procedures and greens represent decision nodes with removed procedures in panels **b** and **c**. M and F represent male and female gender, respectively, followed by patient age. **•** denotes a patient who was subsequently diagnosed with urothelial carcinoma of the bladder. Horizontal black lines indicate test result subgroups. The vertical green bar indicates Cxbladder Triage negative and Cxbladder Detect normal results. The vertical blue bar indicates Cxbladder Triage physician directed protocol results. The vertical red bar in panel **b** indicates Cxbladder Triage physician directed protocol results and in panel **c** indicates results that are also Cxbladder Detect positive

**Fig. 3:**
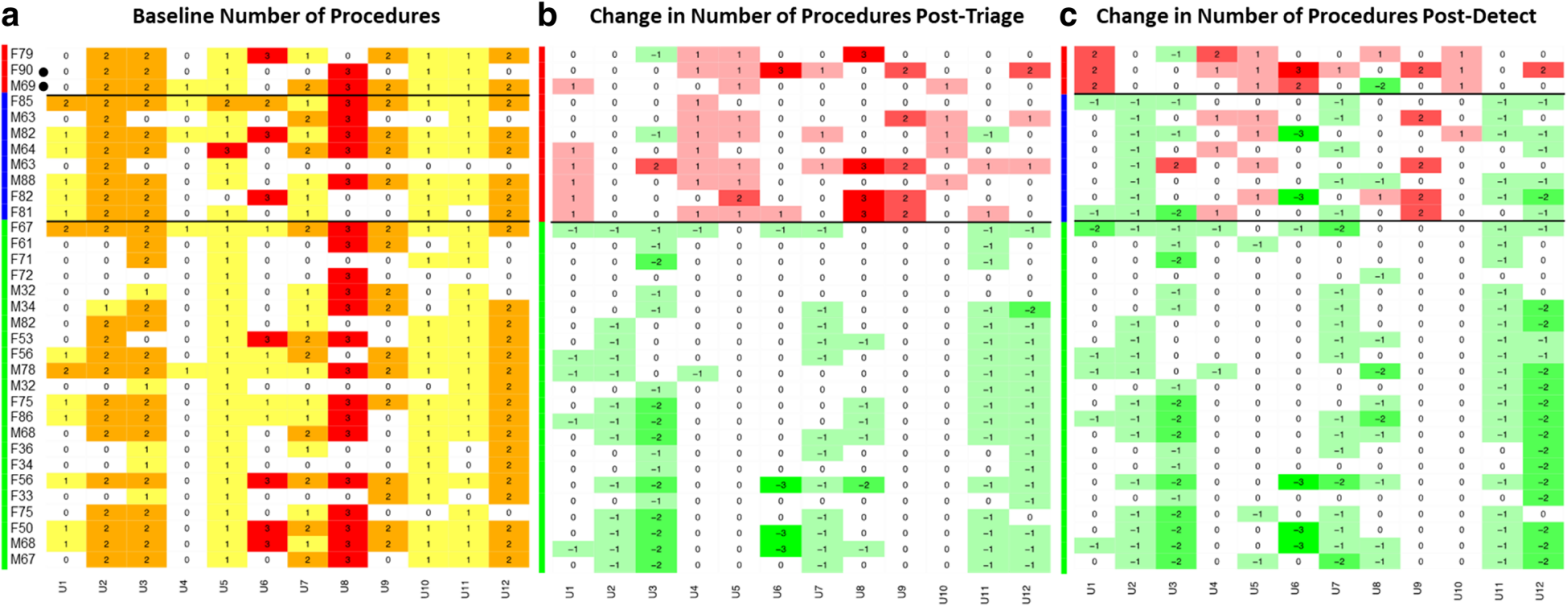
Heat maps representing the number of invasive diagnostic tests. Panel **a**: Baseline number of procedures; Panel **b**: Change from baseline after presenting the results of Cxbladder Triage; Panel **c**: Change from baseline after presenting the results of Cxbladder Triage and Detect. Columns represent participant physicians. Rows represent patients. Each cell represents a patient-physician decision node. Reds represent decisions nodes with added procedures and greens represent decision nodes with removed procedures in panels **b** and **c**. M and F represent male and female gender, respectively, followed by patient age. **•** denotes a patient who was subsequently diagnosed with urothelial carcinoma of the bladder. Horizontal black lines indicate test result subgoups. The vertical green bar indicates Cxbladder Triage negative results and Cxbladder Detect normal results. The vertical blue bar indicates Cxbladder Triage physician directed protocol results. The vertical red bar in panel **b** indicates Cxbladder Triage physician directed protocol results and in panel **c** results that are also Cxbladder Detect positive

A total of 688 diagnostic procedures were requested by participating physicians across 278 baseline clinical decisions (Fig. 2a), including 424 invasive procedures in 259 clinical decisions (Fig. 3a). No additional diagnostic procedures were requested in 118 baseline clinical decisions.

### Change in procedure selection following a negative result for Cxbladder triage

Negative Cxbladder Triage results led to a net reduction in procedures in 79/182 (43.4%) clinical decisions where additional diagnostic procedures had been requested based on baseline data (Fig. 2a+b). This translated into a significant mean reduction in total diagnostic procedures requested per interaction (Table 1). Only one patient with a negative result had a net increase in procedures (Fig. 2b).

**Table 1:** Mean absolute and proportional change in the use of diagnostic tests per patient

	Negative		Physician-directed Protocol /Normal		Physician-directed Protocol /Positive	
	Δ (95% CI)	Δ%	Δ (95% CI)	Δ%	Δ (95% CI)	Δ%
Total	−0.686 (− 0.854, − 0.517)*	−41	− 0.208 (− 0.488, 0.072)	−11	0.722 (0.265, 1.179)*	+ 38
Invasive	−0.519 (− 0.639, − 0.399)*	−51	− 0.198 (− 0.397, 0.001)	−17	0.639 (0.314, 0.963)*	+ 55
Cystoscopy						
Flexible	−0.352 (− 0.422, − 0.283)*	− 56	0.187 (− 0.303, − 0.072)	−28	0.000 (− 0.188, 0.188)	0
Rigid	0.000 (− 0.021, 0.021)	0	0.031 (− 0.004, 0.067)	+ 300	0.250 (0.192, 0.308)*	+ 900
CT scan						
Contrast	−0.155 (− 0.214, − 0.096)*	−48	−0.031 (− 0.129, 0.067)	−8	0.361 (0.201, 0.521)*	+ 100
Non-contrast	−0.011 (− 0.043, 0.020)	−16	−0.010 (− 0.063, 0.042)	−13	0.028 (− 0.058, 0.113)	+ 25
Non-invasive tests						
Ultrasound	0.034 (−0.022, 0.090)	+ 11	0.083 (−0.009, 0.176)	+ 26	− 0.056 (− 0.207, 0.096)	−18
Urine cytology	− 0.170 (− 0.229, − 0.112)	− 60	− 0.062 (− 0.159, 0.034)	−18	0.167 (0.009, 0.325)*	+ 43
UroVysion® FISH	−0.030 (− 0.051, − 0.009)*	−100	−0.031 (− 0.066, 0.004)	− 75	−0.028 (− 0.085, 0.029)	−50

*p < 0.05

Abbreviations: *CT* computed tomography, *FISH* fluorescence in situ hybridization

Presentation of negative results from Cxbladder Triage resulted in a net removal of invasive procedures in 93/169 clinical decisions (55.0%) where invasive procedures had been requested at baseline (Fig. 3b). Of the flexible cystoscopies and contrast CT scans requested for this group, 40.4% (67/166) and 46.5% (40/86), respectively, were abandoned (Additional file 2: Figure S1 and Additional file 3: Figure S2).

### Change in procedure selection following a physician-directed protocol from Cxbladder triage and positive result for Cxbladder detect

Following presentation of results from Cxbladder Detect for patients who were guided to a physician-directed protocol by Cxbladder Triage, 19/36 clinical decisions (52.8%), where a patient tested positive, resulted in a net increase in procedures, while three clinical decisions (8.3%) resulted in a net decrease (Fig. 2c). Overall, a positive result for Cxbladder Detect resulted in a significant increase in the total number of procedures per interaction (Table 1).

A net 26 invasive procedures were added across 17 clinical decisions for patients who tested positive using Cxbladder Detect, while three procedures were removed in a further three clinical decisions (Fig. 3c). No net change in the number of clinical decisions involving a request for a flexible cystoscopy occurred in this group (Additional file 2: Figure S1), but 9 additional rigid cystoscopies were requested. Furthermore, a net 13 clinical decisions resulted in the addition of a contrast CT scan (Additional file 3: Figure S2). One extra non-contrast CT scan was also requested.

### Clinical decisions involving a physician-directed protocol from Cxbladder triage and normal result for Cxbladder detect

Of the 96 clinical decisions resulting in a physician-directed protocol that subsequently tested normal for Cxbladder Detect, 31 had a net increase, and 17 a net decrease in total procedures (Fig. 2c). However, a normal result from Cxbladder Detect was associated with a significant mean reduction in total diagnostic procedures requested per patient interaction versus baseline (Table 1).

A net reduction in invasive procedures compared with baseline occurred in 32/66 (48.5%) clinical decisions following a normal result from Cxbladder Detect, while procedures were added in 14/96 (14.6%) clinical decisions (Fig. 3c). Overall, 28 (42.4%) fewer flexible cystoscopies were requested in this group (Additional file 2: Figure S1), but three rigid cystoscopies were added. Three fewer contrast CT scans (8.1% reduction versus baseline) and one less non-contrast CT scan were requested (Additional file 3: Figure S2).

### Transition probability analyses

**Fig. 4:**
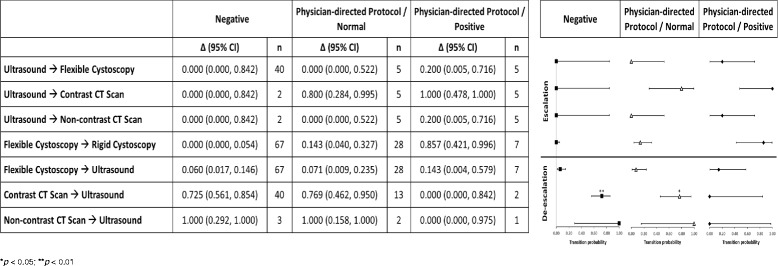
Transition probabilities for diagnostic tests following disclosure of results from Cxbladder Triage and Detect. *p < 0.05; **p < 0.01

Transition probability analyses indicate a de-escalation of procedures following a negative result from Cxbladder Triage (Fig. 4). For example, 72.5% (29/40) of patients with negative results who were allocated a contrast CT scan at baseline had this procedure substituted with ultrasound.

Transition analyses also indicate escalation of diagnostic procedures for patients following a physician-directed protocol result (Fig. 4). All five patients with these results who were allocated an ultrasound at baseline had this procedure substituted with a contrast CT scan. Six out of seven (85.7%) patients allocated a flexible cystoscopy at baseline were escalated to rigid cystoscopy.

## Discussion

The assessment process undertaken in this study with participating physicians retrospectively evaluating real world patients extends the previous study [12] by demonstrating that the intensity and focus of the physician recommended workup is influenced by the Cxbladder result without compromising detection of UC. The heatmap data graphic was developed to enhance the visualization of the changes in decisions for the evaluation of these patients, as either an escalation or de-escalation from baseline decisions, after the addition of the Cxbladder test results. This approach provided the opportunity to view changes in decision making and their impact on clinical utility at the level of the individual physician-patient interaction as opposed to the consolidated cohort-wide view of our previous study [12].

The addition of non-invasive Cxbladder urine biomarker test results to the physicians decision making process was shown in this study to provide an overall increase in clinical utility of Cxbladder as an objective risk assessment tool for UC in the work-up of patients presenting with AMH where there are no obvious benign causes. More specifically, presentation of Cxbladder results leads to physicians modifying their diagnostic behavior and resource allocation in a consistent and repeatable manner reflecting the risk of disease for the patient. In addition, 33% (8/24) of the clinical decisions where the patient had UC, would not have been referred for an appropriate work-up that included invasive diagnostics tests in the absence of results from Cxbladder [12].

The addition of Cxbladder test results for decision making was shown to enable patients with a high probability of UC to be identified and prioritized for a full urological work-up in advance of an initial consultation, offering a clear increase in clinical utility. This outcome is expected to be of significant importance in a healthcare system when there are challenges from < 14% of patients with hematuria being referred to a urologist within 180 days [15], and where approximately 20,000 instances of UC are missed annually in the US amongst patients with hematuria who are considered to be moderate-to-high risk [14]. Furthermore, approximately, one-third of patients with hematuria undergo a cystoscopy, providing the potential for approximately 200,000 potentially unnecessary cystoscopies performed on patients who have a low risk of UC every year [14]. Accordingly, Cxbladder has the potential to provide an enhancement to the standard of care for the management and diagnostic workup of patients with hematuria that will provide benefits to the patient and the healthcare system alike from the appropriate targeting and reduction in the total number and invasiveness of tests and procedures used.

The addition of Cxbladder to the decision making by participating physicians evaluating the retrospective patient data showed an increase in the clinical utility from a reduction in the overall diagnostic burden of patients with hematuria. The previous study provided a population-level perspective and demonstrated a reduction in the total number and invasive procedures by 25% and 31%, respectively [12]. The present study extends the analysis at the individual physician-patient level with repeatable outcome results. Specifically the results identified that all participant physicians modified their chosen diagnostic work-ups to reflect the change in risk assessment provided by the addition of Cxbladder test results for each patient, i.e. an escalation or de-escalation of the number and invasiveness of the work-ups appropriately reflecting the change in the probability of UC. Notably, this was often observed as a change in nature or type of the tests and procedures requested, not only the total number. In particular, the prevalence of UC was 3-fold higher in the subset of patients referred for a physician-directed protocol by Cxbladder Triage, providing evidence that the escalation of procedures is appropriate for this subset of patients, while no patients with a negative result had UC, justifying a de-escalation.

Evidence from this study shows that the provision of more objective data on the probability of UC does not result in harm to patients, i.e. a reduced risk of patients who were ultimately diagnosed with UC in the real world not undergoing an appropriate urological work up [12]. On the contrary, more information on the risk of UC is likely to lead to a lowering of the level of variance observed during the diagnostic process to the benefit of patients; high-risk patients may be prioritized, while less invasive diagnostic tests and procedures may be recommended for low-risk patients [12].

The present data provide evidence that the use of a quantitative tool can guide the selection of diagnostic procedures for up to 20% of the general patient population with AMH [14, 15, 18, 20, 25]. Indeed, presenting Cxbladder test results to urologists led to all patients with UC being allocated guideline-recommended procedures compared with 2/3 of patients with baseline referral data alone [12].

Invasive procedures have continued to be relied upon by physicians because, as noted by the US Centers for Medicaid and Medicare [28], studies directly correlating the results of molecular diagnostics with clinical outcomes are lacking. The field of molecular diagnostics has promised to offer superior outcomes compared with invasive options for the patient, healthcare organizations and the wider society, but earlier iterations of non-invasive urine tests remain absent from diagnostic algorithms for patients with hematuria because of their low clinical resolution and lack of demonstrated clinical utility.

Accordingly, routine use of Cxbladder would likely reduce the burden of care for both patients and healthcare systems through the more selective use of invasive procedures, particularly CT scans, without compromising the desire of patients and physicians to achieve direct visualization and identification of any tumor as part of the diagnostic process [29]. This is consistent with suggestions made by Halpern and colleagues (2017) [2] regarding the cost-effective use of diagnostic procedures in patients with AMH, while also addressing criticism of the clinical implications of advocating for a blanket replacement of CT scans with ultrasound [30]. Furthermore, the utility of Cxbladder as a risk assessment tool is likely to be further enhanced by introduction of the Cxbladder Resolve test as a third step for patients with physician-directed protocol result that identifies patients with a high probability of high-grade UC [31].

### Strengths and weaknesses

In the absence of clear guidance or precedents for assessing the clinical utility of non-invasive diagnostic tests, the investigators considered the study design used here to be the most pragmatic method of evaluating the clinical utility of Cxbladder in a cohort of real-world patients presenting with AMH given the logistical, consistency and ethical challenges in prospectively investigating the selection of diagnostic tests in patients with AMH. Notably, the innovative methodology applied in this study acknowledges and addresses the concerns expressed in the literature by experts in the field regarding the potential risks of using non-invasive urine tests for UC in patients with AMH without risking patient health. Furthermore, the methodology has allowed the challenges in weighing the risk-benefit profile for individual patients using clinical judgement alone to be identified. Accordingly, this methodology has provided hypothesis-generating data to justify further clinical investigation into the use of the new and more sensitive, non-invasive tests such as Cxbladder in increasing clinical resolution of patients being worked up who have presented to the clinic with hematuria.

The design, by requiring each physician to evaluate all patients, takes out ‘patient’ as a variable, thereby permitting focus on the physician-patient interaction, whose focus is the main aim of the study.

While cases were systematically selected, to provide a broad representation of patients in the prospective studies, data was presented immediately and across a series of patients: the investigators acknowledge that the study may not be reflective of a true clinical scenario. The influence of pre- and intra-study experience influencing decision-making cannot also be ruled out. However, by using the same patients presented to all urologists the study removed any confounding effect caused by different patients for different urologists and accordingly the study measured urologist decision making independent of patient variance. Furthermore, the outcomes of this study have not yet been tested in the real-world where outside influences, such as patient selection, resource availability, costs, alternative diagnostic options and real patient outcomes could influence the translation of these outcomes into clinical practice.

## Conclusion

The non-invasive Cxbladder urine test is being used by physicians to objectively define the risk of UC in patients with AMH with sufficient resolution to guide more efficient diagnostic procedure selection and resource allocation to the benefit of both patients and healthcare systems, particularly underserved groups, such as women, elderly patients and younger patients. The increase in clinical resolution associated with Cxbladder has the potential to significantly improve the identification of patients with UC when compared to the tests and procedures selected in the more conventional or standard work-up without compromising patient safety. Any consequential reduction in total and invasive procedures, coupled with the increased resolution provided by Cxbladder, would minimize the risk of invasive test-related harm and provides an increase in net patient benefit. Translation of the observations reported here into clinical outcomes would simplify the clinical algorithms applied when working up patients with AMH improving outcomes for patients, physicians and healthcare systems.

## Additional files

Additional file 1: **Table S1.** Participating physicians. (DOCX 12 kb)

Additional file 2: **Figure S1.** Heat maps representing the number of flexible cystoscopies^a,b^. (DOCX 237 kb)

Additional file 3: **Figure S2.** Heat maps representing the number of contrast CT scans^a,b^. (DOCX 463 kb)

## Abbreviations

AMH: Asymptomatic microscopic hematuria
AUA: American Urological Association
CI: Confidence interval
CT: Computed tomography
UC: Urothelial carcinoma
