# Supplementary material for: Clinical utility of a non-invasive urine test for risk assessing patients with no obvious benign cause of hematuria: a physician-patient real world data analysis
Source: BMC Urol. 2018 Mar 9;18:18. doi: 10.1186/s12894-018-0327-6 (PMC5845194; doi:10.1186/s12894-018-0327-6)
Supplement: Supplementary file 1 — Table S1. Participating physicians. (DOCX 12 kb) [file 12894_2018_327_MOESM1_ESM.docx]

**Table S1.** Participating physicians

| **Physician** | **Affiliation** |
| --- | --- |
| Lewis Chan | Concord Hospital, Sydney, NSW, AUSTRALIA |
| Christophe Chemasle | Palmerston North Urology, Palmerston North, NEW ZEALAND |
| Joe DiTrolio | Roseland, NJ, USA |
| Sia Daneshmand | University of Southern California, Los Angeles, CA, USA |
| Peter Gilling | Urology BOP, Tauranga, NEW ZEALAND |
| Dennis Gyomber | John Fawkner Hospital Consulting Suites, Melbourne, VIC, AUSTRALIA |
| Jonathan Masters | Auckland City Hospital, Auckland, NEW ZEALAND |
| Scott Owens | Urology of Central Pennsylvania, Camp Hill, PA, USA |
| Jay Raman | Penn State Milton S. Hershey Medical Center, Hershey, PA, USA |
| Rod Studd | Southern Cross Hospital, Wellington, NEW ZEALAND |
| James Sylora | Mid West Urologist, Evergreen Park, IL, USA |
| Kian Tai | Tan Tock Seng Hospital Medical Centre, SINGAPORE |
