# Supplementary material for: Clinical utility of a non-invasive urine test for risk assessing patients with no obvious benign cause of hematuria: a physician-patient real world data analysis
Source: BMC Urol. 2018 Mar 9;18:18. doi: 10.1186/s12894-018-0327-6 (PMC5845194; doi:10.1186/s12894-018-0327-6)
Supplement: Supplementary file 2 — Figure S1. Heat maps representing the number of flexible cystoscopiesa,b. (DOCX 237 kb) [file 12894_2018_327_MOESM2_ESM.docx]

**Supplementary Materials**

**Figure S1.** Heat maps representing the number of flexible cystoscopies^a,b^

**
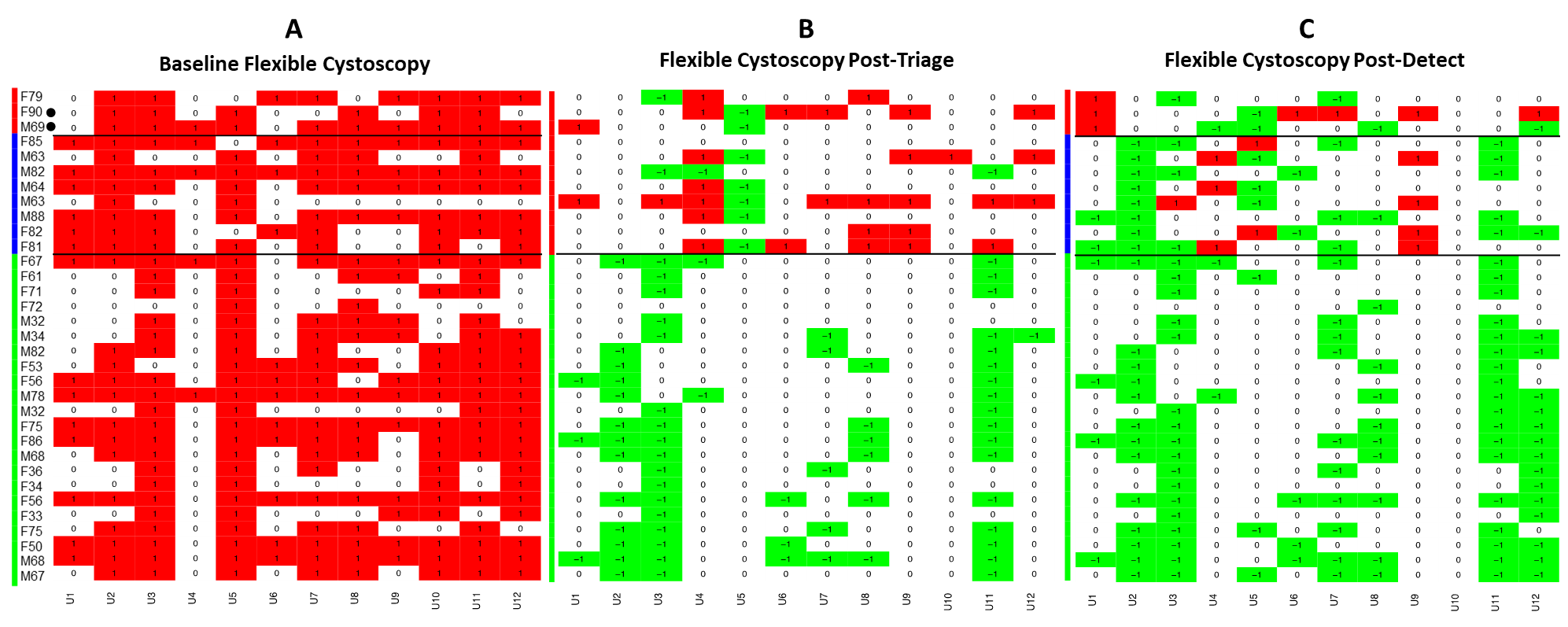
**

^a^Panel A: Baseline number of procedures; Panel B: Change from baseline after presenting the results of Cxbladder Triage; Panel C: Change from baseline after presenting the results of Cxbladder Triage and Detect.

^b^Columns represent participant physicians. Rows represent patients. Each cell represents a patient-physician decision node. Reds represent decisions nodes with added procedures and greens represent decision nodes with removed procedures in panels B and C. M and F represent male and female gender, respectively, followed by patient age. • denotes a patient who was subsequently diagnosed with urothelial carcinoma of the bladder.
